# Feasibility of imaging synaptic density in the human spinal cord using [^11^C]UCB-J PET

**DOI:** 10.1186/s40658-022-00464-0

**Published:** 2022-05-03

**Authors:** Samantha Rossano, Takuya Toyonaga, Jason Bini, Nabeel Nabulsi, Jim Ropchan, Zhengxin Cai, Yiyun Huang, Richard E. Carson

**Affiliations:** 1 Department of Radiology and Biomedical Imaging, Yale PET Center, Yale School of Medicine, P.O. Box 208048, New Haven, CT 06520 USA; 2 Department of Biomedical Engineering, Yale University, New Haven, CT USA

**Keywords:** Synaptic density, SV2A, Spinal cord, PET

## Abstract

**Purpose:**

Neuronal damage and synapse loss in the spinal cord (SC) have been implicated in spinal cord injury (SCI) and neurodegenerative disorders such as Amyotrophic Lateral Sclerosis (ALS). Current standards of diagnosis for SCI include CT or MRI imaging to evaluate injury severity. The current study explores the use of PET imaging with [^11^C]UCB-J, which targets the synaptic vesicle protein 2A (SV2A), in the human spinal cord, as a way to visualize synaptic density and integrity in vivo.

**Results:**

First, simulations of baseline and blocking [^11^C]UCB-J HRRT scans were performed, based on SC dimensions and SV2A distribution to predict *V*_T_, *V*_ND_, and *V*_S_ values. Next, human baseline and blocking [^11^C]UCB-J HRRT images were used to estimate these values in the cervical SC (cSC). Simulation results had excellent agreement with observed values of *V*_T_, *V*_ND_, and *V*_S_ from the real human data, with baseline *V*_T_, *V*_ND_, and *V*_S_ of 3.07, 2.15, and 0.92 mL/cm^3^, respectively, with a *BP*_ND_ of 0.43. Lastly, we explored full SC imaging with whole-body images. Using automated SC regions of interest (ROIs) for the full SC, cSC, and thoracic SC (tSC), the distribution volume ratio (DVR) was estimated using the brain gray matter as a reference region to evaluate SC SV2A density relative to the brain. In full body imaging, DVR values of full SC, cSC, and tSC were 0.115, 0.145, and 0.112, respectively. Therefore, measured [^11^C]UCB-J uptake, and thus SV2A density, is much lower in the SC than in the brain.

**Conclusions:**

The results presented here provide evidence for the feasibility of SV2A PET imaging in the human SC, however, specific binding of [^11^C]UCB-J is low. Ongoing and future work include further classification of SV2A distribution in the SC as well as exploring higher-affinity PET radioligands for SC imaging.

**Supplementary Information:**

The online version contains supplementary material available at 10.1186/s40658-022-00464-0.

## Background

The central nervous system (CNS) is comprised of the brain and the spinal cord and is responsible for transmitting nerve signals that control critical functions in the body, such as locomotion and respiration. The spinal cord (SC) has a key role in relaying nerve signals between the periphery and the brain, and also can respond to external stimuli by spinal reflex. The integrity of SC neurons and synapses is crucial for proper function, and traumatic spinal cord injury (SCI) or neurodegenerative diseases may lead to interruption or loss of these functions. Specifically, physical damage to the SC in traumatic SCI disrupts neural circuits and in turn results in loss of functions that depend on the location of the lesion. Studies have shown that functional recovery may be possible, depending on the severity of the injury [1, 2]. Synapse loss in the spinal cord has also been suggested in the pathology of amyotrophic lateral sclerosis (ALS), a neurodegenerative disease that causes dysfunction and eventually total loss of function in neural circuits crucial for movement, speech, and breathing [3]. Additionally, synapse loss in the spinal cord has been shown in mouse models of spinal muscular atrophy, an autosomal recessive disease that causes atrophy and muscle weakness resulting from motor neuron death[4]. Synaptic pathologies of these conditions are typically studied in animal models, as there have been no methods to study synaptic density in vivo.

Clinical imaging techniques, such as computed tomography (CT) and magnetic resonance imaging (MRI), have been studied for evaluation of spinal cord conditions [5–7]. These methods aid in visualizing and assessing the degree of structural lesions, and functional MRI techniques can explore the physiological function and protein composition of the tissue surrounding the site of injury. Positron emission tomography (PET) is a functional imaging modality which quantifies physiological properties of tissue in vivo. Synaptic vesicle glycoprotein 2A (SV2A) is the target of interest for novel PET radiotracers, and imaging with this ligand can assess synaptic density in the living brain [8], as SV2A exists in presynaptic vesicles ubiquitously across the brain [9]. [^11^C]UCB-J, a SV2A PET radioligand, has been used to explore SV2A differences in clinical populations including epilepsy [10], depression [11], and Alzheimer’s disease [12, 13]. Thus, measures from SV2A PET are sensitive to synaptic loss and pathologies in various brain diseases and disorders.

Although SV2A as a marker for synaptic density in the brain has been studied robustly, SV2A as a marker for synaptic density in the spinal cord has not been well established. Previous studies report SV2A expression in the rodent spinal cord, at concentrations 2-to-fivefold lower than the cortex [14]. Since SV2A is expressed in the mammalian spinal cord, it is possible that the full CNS may be of potential interest for SV2A PET imaging studies. However, this is not without challenges. Given the limitations of whole body PET imaging, such as poor spatial resolution that contributes to partial volume effects (PVE), small structures and lower concentration of protein in the spinal cord, and the dependence on high affinity radioligands for quantitative accuracy, PET imaging in the spinal cord remains an area for which more optimization is needed before it can be utilized as a clinical tool. If useful, SV2A PET imaging in the spinal cord can provide ways to evaluate synaptic integrity of patients with SCI, ALS, or other neurodegenerative disorders, and can aid in diagnosis and prognosis of these conditions, along with potential utility in assessing treatment efficacy, based on functional synapse density.

The current study explores the use of [^11^C]UCB-J PET for in vivo evaluation of synaptic density in the spinal cord by imaging SV2A in human spinal cord. Firstly, a simulation study was completed to model the capability of imaging SV2A in the spinal cord at the resolution of the high-resolution brain PET scanner, the High-Resolution Research Tomograph (HRRT). Next, human baseline and blocking [^11^C]UCB-J images acquired on the HRRT were used to evaluate SV2A blocking and *V*_ND_ in the cervical spinal cord. Lastly, total body images aided in the visualization of [^11^C]UCB-J distribution in the full spinal cord, and was quantified using the simplified reference tissue model (SRTM) to generate distribution volume ratios (DVR) of spinal cord radioactivity in relation to brain gray matter binding.

## Methods

### Simulation of SV2A in the spinal cord

A simulation study was designed to evaluate the ability of using [^11^C]UCB-J PET to measure SV2A blocking and *V*_ND_ in the spinal cord (SC) at a resolution comparable to the Siemens High Resolution Research Tomograph (HRRT). Prior studies have shown that SV2A concentrations are 3–to-fivefold lower in the spinal cord (SC) than in the cortex [14]. Knowing that the PET outcome measure of total volume of distribution is the total of the nondisplaceable PET binding and the specific PET binding (*V*_T_ = *V*_ND_ + *V*_S_), we can simulate the *V*_T_ in the gray matter (GM), white matter (WM) and full SC using the following assumptions: 1) as *V*_S_^SC^ = 0.2 * *V*_S_^Cortex^, 2) volumetrically, the SC is ~ 75% WM and ~ 25% GM [15], 3) *V*_S_ is minimal in the WM (~ 0.00 mL/cm^3^), and 4) *V*_ND_ is the same across brain and SC GM at approximately 2.8 mL/cm^3^ [16]. Prior studies suggest that *V*_ND_ in the white matter is slightly greater than in the GM, at about 3.3 mL/cm^3^, however, for this simulation, *V*_ND_ was assumed to be uniform across gray and white matter. The assumptions and definitions of the parameters in this simulation are outlined in Table 1. *V*_T_ images of the SC under baseline and blocking with 75% receptor occupancy (*r*) conditions were generated with the full spinal cord as an ellipse with radii of 3 voxels (~ 3.6 mm) and 5 voxels (~ 6.1 mm). Voxels were assigned as white or gray matter to mimic the cord, with gray matter centered. Simulated baseline and blocking images were blurred using a Gaussian filter (4 mm FWHM), which is the estimated resolution at the edge of the axial field of view of the HRRT, based on estimations from line source phantom studies (data not shown). The simulated *V*_T_ values under baseline and blocking conditions were calculated for the full SC ROI and for voxels outside the spinal cord (non-cord), and observed *V*_ND_ and *V*_S_ values for the full SC were calculated as described in Eqs. 1 and 2, derived from the occupancy plot equation [17], where *r* is assumed known:

1 $$V_{{{\text{ND}}}}^{{{\text{Observed}}}} = \frac{{(V_{{\text{T}}}^{{{\text{Baseline}}}} - V_{{\text{T}}}^{{{\text{Blocking}}}} ) - r*V_{{\text{T}}}^{{{\text{Baseline}}}} }}{r}$$

2 $$V_{{\text{S}}}^{{\text{Observed }}} = V_{{\text{T}}}^{{{\text{Baseline}}}} - V_{{{\text{ND}}}}^{{{\text{Observed}}}}$$

**Table 1:** Table of definitions and assumptions for simulation study

Parameter definition	Cortex	Spinal cord (SC) gray matter (GM)	Spinal cord (SC) white matter (WM)	Full spinal cord (SC) ~ 3:1 WM:GM [15]
Relative SV2A concentration, based on ex vivo SV2A concentrations [14]	1.00	0.20	0.00	
*V*_ND_, Nondisplaceable Volume of Distribution	2.80	2.80	2.80	2.80
*V*_S_, Specific Volume of Distribution of SV2A	17.2	3.44	0.00	0.81
Baseline *V*_T_, Total Volume of Distribution = *V*_ND_ + *V*_S_	20.0^a^	6.24	2.80	3.83
Blocking *V*_T_ (75% occupancy), Total Volume of Distribution = *V*_ND_ + (1 − r)*V_S_	7.10	3.66	2.80	3.06

^a^Approximate mean GM *V*_T_[19]

### Cervical spinal cord imaging on the HRRT

Four human research participants (2 M/2F, 24–46 years) completed PET scanning with [^11^C]UCB-J on the Siemens HRRT under baseline conditions and blocking conditions with either Levetiracetam (LEV) or Brivaracetam (BRV). These scans were selected from a cohort of subjects previously published [16, 18], based on the positioning of the head in the scanner, such that both baseline and blocking images included at least ~ 20 mm (> 20 axial slices) of cervical spinal cord at the end of the FOV. Arterial blood sampling, PET data collection and image reconstruction were completed as described previously [16, 18]. On an early summed PET image (0–10 min), the location of the cervical spinal cord (cSC) was manually defined, and a cylindrical ROI with radius = 3 voxels (~ 4 mm) was generated along 15 axial slices (~ 18.5 mm). The cSC ROI for a representative subject is shown in Additional file 1. Time activity curves (TACs) of this region were fit with a 1-tissue compartment model (1TCM) to estimate *V*_T_ in the cSC in baseline and blocking scans. Parametric images of *V*_T_ were generated by voxel-wise fitting of the 1TCM as described previously [19]. For each subject, regional brain *V*_T_ estimates from both baseline and blocking scans were used to estimate SV2A occupancy using the Lassen Occupancy Plot [17]. The observed occupancy from the Lassen plot was used along with the baseline and blocking cSC *V*_T_ in Eqs. 1 and 2 to calculate observed *V*_ND_ and *V*_S_ in the cSC.

### Full spinal cord imaging on the mCT

PET scans used for this portion of the study were acquired and published for dosimetry purposes previously [20]. Four human research participants (2 M, 2F, 26–47 years) completed PET/CT scanning on the Siemens mCT with [^11^C]UCB-J as described previously [20]. Arterial blood sampling was not performed. CT images were acquired prior to PET scanning and were used for attenuation correction and scatter correction [21], as well as ROI definition. Dynamic PET scanning with [^11^C]UCB-J was completed using continuous bed motion with the following frame times: 4 × 60 s, 2 × 120 s, 23 × 5 min. PET images were reconstructed using time of flight (TOF) and point spread function (PSF) modeling and ordered subset expectation maximization (OSEM) algorithm with 2 iterations, 21 subsets (Siemens Medical Solutions, USA). A summed standardized uptake value (SUV) image (30–60 min) was used to threshold brain uptake to define a gray matter (GM) ROI (MR images were not available).

The spinal cord ROI was defined using the CT image. Firstly, the CT image was cropped to include the SC, starting from the base of the skull to the L2/L3 vertebrae. The voxels with intensities similar to bone were binarized into a mask. This spine mask was closed to fill holes within and between vertebrae by dilating and eroding the binary mask with a kernel with radius of 5 voxels. The Center of Mass in x (COMx) and y (COMy) directions were calculated on each axial slice, and a cubic spline was used to fit COMx and COMy. Then, on each axial slice, a circular ROI with radius of 2 voxels (~ 4 mm) was generated around this point and a full SC ROI mask was generated. This process is described in Additional file 2. A cervical SC (cSC) was defined from the full SC ROI from the C2 to C6 vertebrae. A thoracic SC (tSC) was defined from the full SC ROI from T2 to T10 vertebrae.

The full SC ROI TAC was fit with the simplified reference tissue model (SRTM2 [22]), using the whole brain GM as a reference region and a fixed ${k}_{2}^{^{\prime}}$ value of 0.018 min^−1^ [19], based on previously published values. Kinetic analysis was completed using 60 min of dynamic PET data for all regional TACs. The distribution volume ratio (DVR) was estimated to explore [^11^C]UCB-J uptake in the SC relative to the brain GM, and should equal *V*_T_^SC^/*V*_T_^GM^.

## Results

### Simulation of SV2A in the spinal cord

**Fig. 1:**
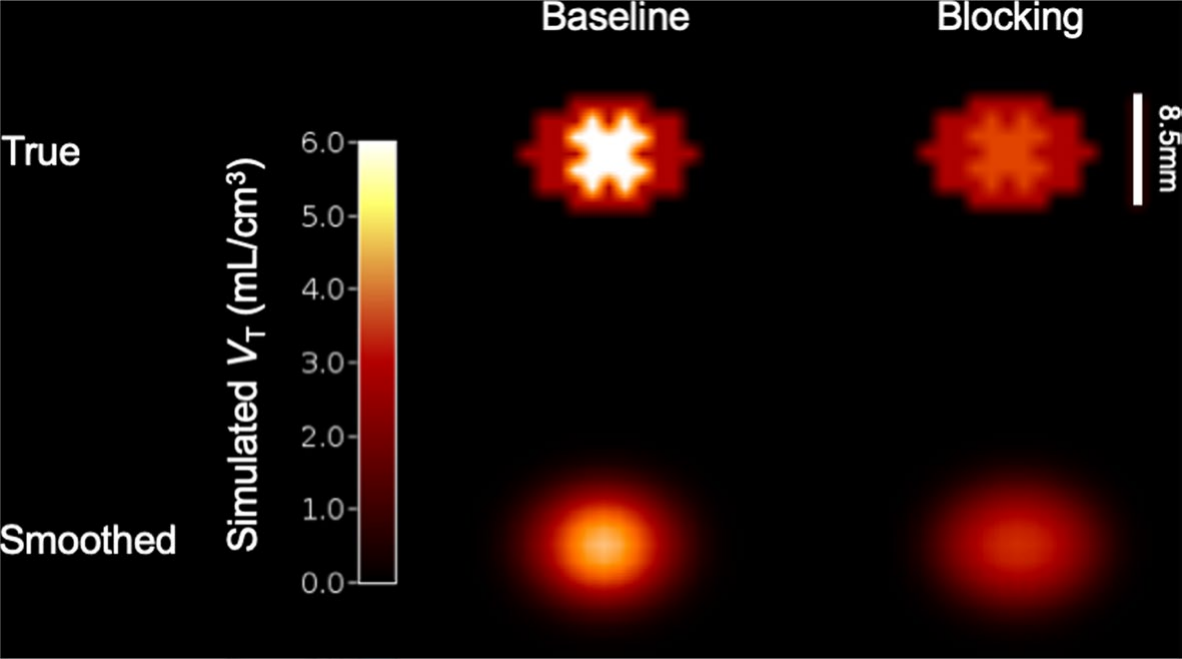
Simulated Spinal Cord images for baseline and blocking conditions. Scale bar = 8.5 mm

Simulated True and Smoothed baseline and blocking images are shown in Fig. 1. The true values of baseline and blocking *V*_T_ values were 3.83 and 3.06 mL/cm^3^, respectively, for the full SC (Table 1), and 0.0 and 0.0 mL/cm^3^ for the non-cord. After smoothing, the measured baseline and blocking *V*_T_ values were 3.09 and 2.36 mL/cm^3^, respectively, for the full SC and 0.02 mL/cm^3^ and 0.02 mL/cm^3^ for the non-cord. With the simulated occupancy of 75%, using Eqs. 1 and 2, the *V*_ND_^observed^ based on these measured ROI estimates is 2.12 mL/cm^3^ and the *V*_S_^observed^ is 0.97 mL/cm^3^.

### Cervical spinal cord imaging on the HRRT

**Fig. 2:**
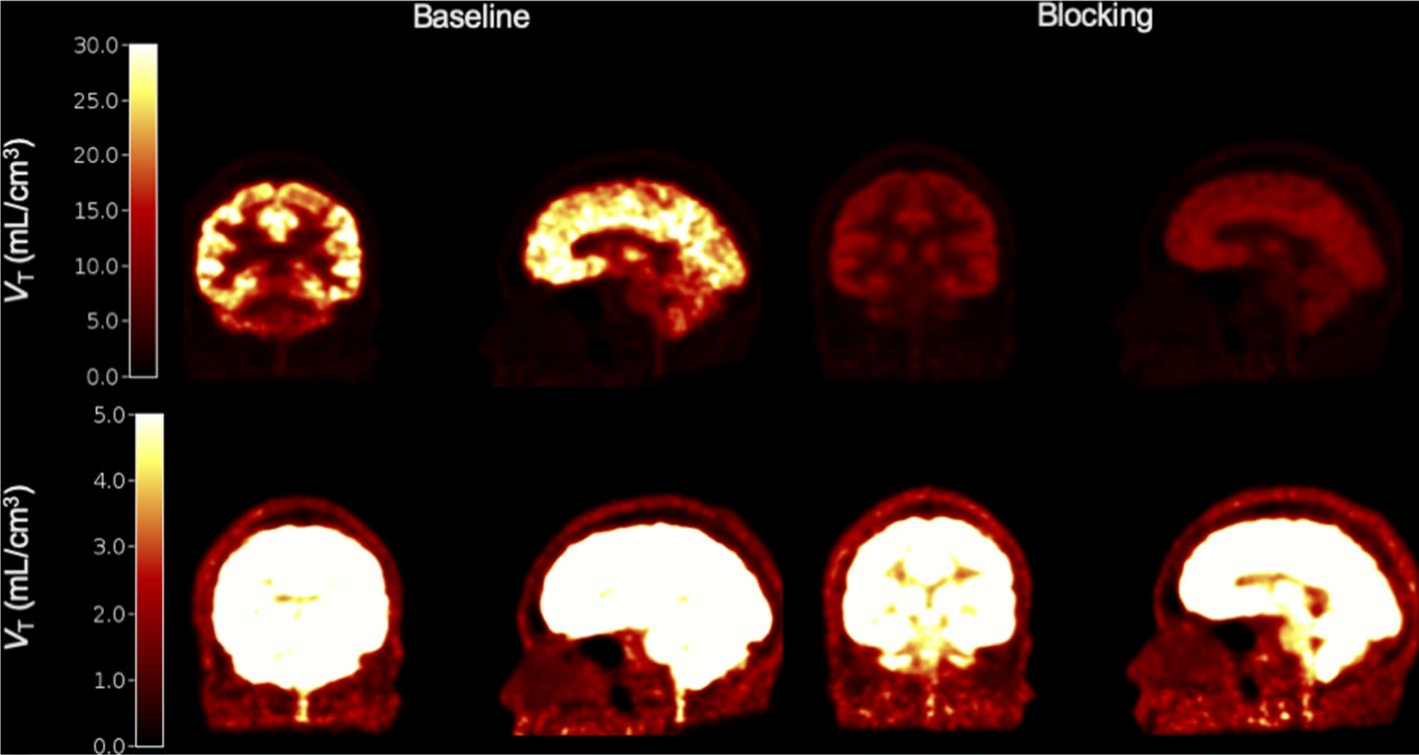
Example baseline (left) and blocking (right) [^11^C]UCB-J *V*_T_ coronal and sagittal images in the whole brain and cSC from the HRRT study. Images are scaled to brain uptake levels in the top row and scaled to cSC uptake in the bottom row

Whole brain and cSC *V*_T_ images from baseline and blocking scans from a representative subject are shown in Fig. 2 in a color scale optimized for whole brain *V*_T_ (top row) and the cSC *V*_T_ (bottom row). Across the four subjects, the mean (s.d., range) brain GM occupancy was 78.4% (8.6%, 65–85%), the mean GM *V*_ND_ was 2.65 mL/cm^3^ (0.39, 2.28–3.08), the mean baseline cSC *V*_T_ was 3.07 mL/cm^3^ (0.87, 2.23–4.29) and the mean blocking cSC *V*_T_ was 2.36 mL/cm^3^ (0.11, 2.22–2.45). The mean *V*_ND_^observed^ was 2.15 mL/cm^3^ (0.24, 1.99–2.50) and the mean *V*_S_^observed^ was 0.92 mL/cm^3^ (1.06, − 0.26–2.30). Thus. the cSC binding potential (*BP*_ND_ = *V*_S_/*V*_ND_) is 0.43. These results strongly agree with the results from the Simulation study above (Table 2). The mean cSC *V*_T_ estimates were 9.1% and 14.6% lower than simulated values at baseline and blocking, respectively. The mean *V*_ND_ observed in the human data was about 16% lower than the simulated results, whereas the mean *V*_S_ was about 13% greater than the simulated results.

**Table 2:** Comparison of outcome measures between simulation study and occupancy study

	Occupancy Study (Mean, *N* = 4)	Simulation study	% Difference, (Occ − Sim)/Sim *100
Baseline *V*_T_ (mL/cm^3^)	3.07	3.09	− 0.6
Blocking *V*_T_ (mL/cm^3^)	2.36	2.36	− 0.2
*V*_ND_ (mL/cm^3^)	2.15	2.12	1.3
*V*_S_ (mL/cm^3^)	0.92	0.97	− 4.7
*BP*_ND_	0.43	0.46	− 6.5

### Full spinal cord imaging in the mCT

**Fig. 3:**
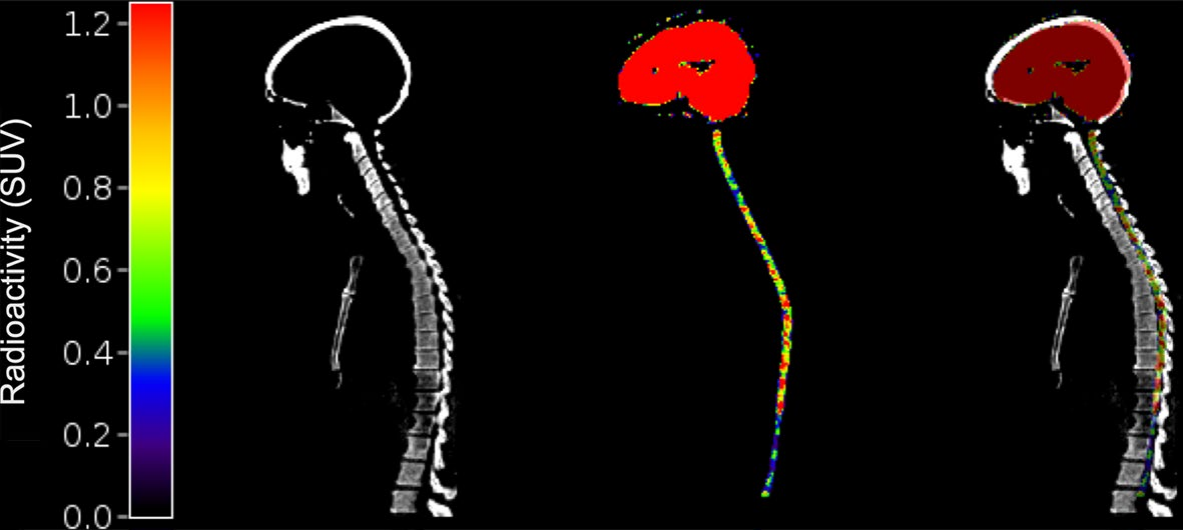
Sagittal view of a CT image, a PET image (SUV, 30–60 min), and overlaid CT/PET of the Full Central Nervous System (brain + SC)

**Fig. 4:**
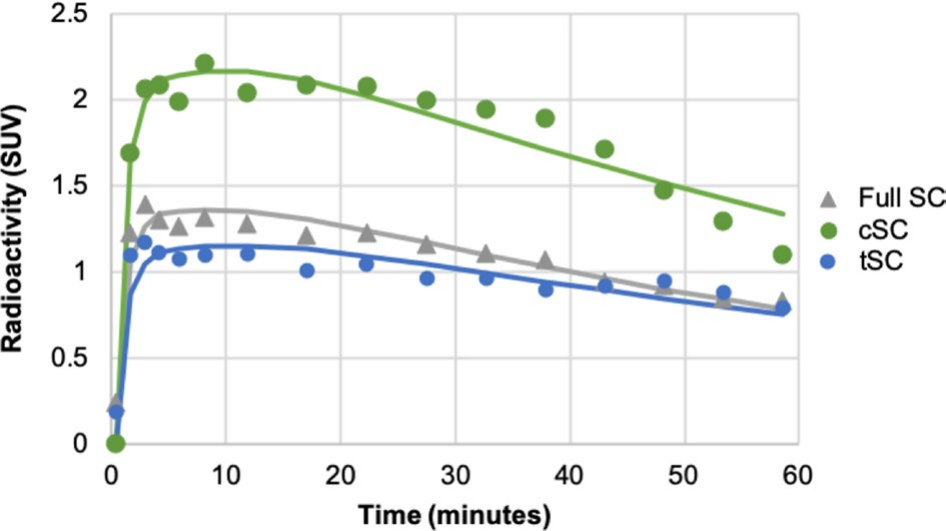
Example full Spinal Cord (SC), cervical spinal cord (cSC), and thoracic spinal cord (tSC) time activity curves with SRTM2 fit (solid line)

An example automated SC ROI is shown in Fig. 3. [^11^C]UCB-J uptake in the SC was 78 ± 5% less than in the whole brain gray matter (peak SUV = 1.9 g/mL in the SC vs. 8.7 g/mL). The SRTM2 fit of the full SC ROI TAC from an example subject is shown in Fig. 4. Using SRTM2, the mean (± SD) DVR (Full SC/brain GM) in the SC was 0.115 (± 0.020). The mean DVR in the cSC was 0.145 (± 0.051) and the mean DVR in the tSC was 0.112 (± 0.016). The individual and mean parameter estimates from SRTM2 for all ROIs are shown in Table 3. DVR represents the ratio of the total volume of distribution between the region of interest and the reference region; in this case, SC DVR = *V*_T_^SC^/*V*_T_^Cortex^. Multiplying the mean DVR in the cSC reported here by the approximate *V*_T_ in the cortex of ~ 20.0 mL/cm^3^, an estimated *V*_T_^SC^ value is 2.90 mL/cm^3^, which is about 5% less than the mean cSC baseline *V*_T_ reported in the human data above, suggesting good agreement in imaging and quantification methods across the two scanners.

**Table 3:** Parameter estimates (%standard error) from SRTM2 fits of automated SC, cSC, and tSC ROI TACs for individual subjects along with mean and SD for 4 subjects

	*DVR*	*R*_1_	*k*_2_
*Full SC*			
Subject 1	0.115 (10.7)	0.231 (8.80)	0.036 (15.7)
Subject 2	0.094 (6.83)	0.268 (6.70)	0.051 (10.6)
Subject 3	0.142 (3.73)	0.204 (2.45)	0.026 (5.31)
Subject 4	0.107 (5.45)	0.150 (3.71)	0.025 (7.77)
Mean (S.D.)	0.115 (0.020)	0.213 (0.049)	0.035 (0.012)
*Cervical SC (cSC)*			
Subject 1	0.143 (7.35)	0.205 (5.20)	0.026 (10.7)
Subject 2	0.074 (7.47)	0.221 (7.30)	0.054 (11.6)
Subject 3	0.181 (10.7)	0.201 (5.89)	0.020 (14.8)
Subject 4	0.183 (4.26)	0.237 (2.77)	0.023 (6.04)
Mean (S.D.)	0.145 (0.051)	0.216 (0.016)	0.031 (0.016)
*Thoracic SC (tSC)*			
Subject 1	0.103 (12.3)	0.213 (10.2)	0.037 (18.1)
Subject 2	0.104 (7.13)	0.293 (7.00)	0.051 (11.1)
Subject 3	0.135 (5.49)	0.210 (3.78)	0.028 (7.87)
Subject 4	0.104 (7.60)	0.124 (4.68)	0.021 (10.7)
Mean (S.D.)	0.112 (0.016)	0.210 (0.069)	0.034 (0.013)

## Discussion

This study explores the feasibility of imaging SV2A in the spinal cord with [^11^C]UCB-J PET. Firstly, baseline and blocking PET data were simulated using previously defined SV2A concentrations in the SC relative to the cortex of ~ 20% [14], conditions of 75% blocking, and the partial volume effect. Given a true *V*_ND_ of 2.8 mL/cm^3^ [16], and assuming no specific binding in the WM, calculated baseline and blocking *V*_T_ values were 3.09 and 2.36 mL/cm^3^, respectively, with an observed *V*_ND_ of 2.12 mL/cm^3^. The simulation suggested that specific binding of [^11^C]UCB-J in the spinal cord is low, with an expected *BP*_ND_ of 0.46. Next, we evaluated specific [^11^C]UCB-J binding in the cSC under baseline and blocking conditions from scans acquired in four human subjects on the HRRT. The observed *V*_T_, *V*_ND_, and *V*_S_ values agreed exceptionally well with the simulated measures, with an expected *BP*_ND_ value of 0.43. Lastly, we explored the feasibility of full SC imaging using whole body [^11^C]UCB-J PET/CT images acquired on the mCT. CT images were used to automatically define ROIs for the full, cervical, and thoracic SC. DVR estimates in these regions show that [^11^C]UCB-J uptake in the full SC relative to cortical GM is 0.115. Interestingly, a higher DVR was observed in the cSC than in the tSC, with DVRs of 0.145 and 0.112, respectively. The DVR in the cSC agreed well with the ratio of baseline *V*_T_ in the SC relative to the brain, with a ratio of approximately 0.154 from the HRRT study. Although the pattern of heterogeneity within the SC has not been well established in the synaptic density literature, it is the same pattern that has been reported in [^18^F]FDG images of the human spinal cord as imaged with PET/CT and PET/MRI [23–26]. The consistency across imaging methods in this study suggests that SV2A may be imaged in the human SC, but [^11^C]UCB-J may not be an ideal PET tracer to use for this purpose, due to its low specific binding.

The current study implemented PET data acquired across two different scanners—the Siemens HRRT for brain/cSC imaging and the Siemens Biograph mCT for full SC imaging—each with different spatial resolutions. For each set of images, the whole SC ROIs were defined in different ways. On the HRRT images, the cSC ROI was defined on an early PET image. On the mCT images, the SC was defined based on the CT image. For both cases, the ROI included the whole SC, consisting of GM in the center of the cord surrounded by WM. A vast majority of the specific SV2A signal is located at the center of the ROI in the GM, with low-to-negligible concentrations of SV2A in the WM. Because of this, the effect of the resolution difference across the two scanners will likely be small, and the PVE caused by the respective spatial resolutions should not drastically affect our quantitation of the SC. Any activity that may spill over from the high activity GM voxels will spill into the relatively low activity WM voxels, which are also included in the SC ROI. The results presented in this work support this premise, particularly in regards to the similarities of SV2A PET measures in the cSC across images of different resolution.

However, PVE may be of importance given different SV2A concentrations and potential differences in radiotracer properties between GM and WM in the SC. The cross section of the SC, including both WM and GM, is elliptical with diameters of about 9 mm in the anteroposterior direction and 12 mm in the transverse direction in the cervical segment and diameters of 6 mm and 9 mm, respectively, in the thoracic segments [27]. Since the human spinal cord is approximately 75% WM by volume [15], if the cross-sectional area of the full cSC is ~ 84.8 mm^2^, then approximately ~ 21.2 mm^2^ or 25% of the full cSC volume will be comprised of GM. Given a PET system with resolution at about 4–5 mm, isolating GM signal from the full cord is impossible without the use of partial volume correction. Of the many advantages to PET/MR imaging in the spinal cord [28], MR images can aid in partial volume correction to isolate PET signals from the spinal cord, the CSF and other surrounding tissues. With fine enough resolution on the MRI, distinction of the GM and WM in the spinal cord may be possible, but this was not feasible with the MRIs acquired for brain images in the current study. In addition, the contrast of GM to WM signal of [^11^C]UCB-J is much lower than what is observed in the brain, due to a lower SV2A concentration in SC, as has been reported in rodents [14]. This lower GM/WM contrast will reduce the potential degree of PVE observed within the SC.

In analyzing the in vivo occupancy study of SV2A in the cSC, the *V*_T_ estimates at baseline and blocking conditions were estimated using 1-tissue compartment model fitting of cSC TACs. ROIs of the cSC were located at the axial edge of the HRRT FOV (since the brain was centered), which has lower sensitivity for gamma ray detection due to smaller solid angle available at this positioning inside the PET scanner. In addition to higher noise, out of field scatter can affect the image quality and quantitation at this location, potentially leading to inaccuracies in the estimates presented here. In the whole-body images, the tSC is particularly sensitive to scatter correction accuracy due to the proximity to the liver, which has very high [^11^C]UCB-J uptake. Inaccurate scatter correction in this region of the image would lead to misestimation of radioactivity concentration in the tSC. In addition to scatter, subject motion may also have an effect on the quantitation of PET radioactivity, particularly in the case of respiratory motion in whole body PET images. Furthermore, using PSF modeling with OSEM image reconstruction methods may increase potential edge or Gibbs artifacts that cause quantitative inaccuracies in PET images, in a manner that is dependent on number of iterations, pixel size, and object size [29]. The effects of these limitations should be explored further, to confirm the current study reports of ~ 20% lower [^11^C]UCB-J DVR in the tSC. In any case, blocking studies in whole body scanning (with arterial blood sampling) may aid in evaluation of specific SV2A binding and *V*_ND_ throughout the full SC.

Although it is part of the central nervous system, the spinal cord differs from the brain particularly in their interaction with circulation. In the brain, the blood–brain barrier (BBB) is a layer of endothelial cells that surround blood vessels in the brain, which have unique characteristics such as lacking fenestrations in cell membranes and tight junctions between cells. The BBB regulates transport of molecules from circulation into brain tissue, particularly keeping larger and more polar particles from entering the brain tissues. Similar to those in brain, blood vessels in the spinal cord have a blood-spinal cord barrier (BSCB), though it is believed that the BSCB may be more permeable than the BBB [30]. This may be of particular interest given that previous studies have suggested that radiometabolites of [^11^C]UCB-J do not pass the BBB [19], but uptake of radiometabolites in the SC tissue is not yet well studied, and may be possible depending on the size or polarity of radiometabolites as well as the permeability of the BSCB. The current study uses 60 min of dynamic PET data to evaluate [^11^C]UCB-J uptake in both cSC images from the HRRT and full SC images on the mCT, which limits the potential effects on the PET measures of radiometabolites, which tend to increase in relative concentration throughout the scan.

Furthermore, there is another difference between the brain and SC that is important to note, especially when using reference tissue methods to quantify PET images of the SC as implemented here. The current study defined a SC ROI that included both GM and WM, while the reference region utilized with SRTM2 was comprised of whole brain GM only. Previous work using [^11^C]UCB-J has reported lower *K*_1_ estimates in WM than in GM regions of the brain [19]. Given that the SC is a majority WM by volume, the *K*_1_ relative to the brain GM, or the *R*_1_ estimate, may be low due to this difference. This is likely the reason why the *R*_1_ estimates that resulted from SRTM2 analysis of the whole-body full SC were low, on the scale of about 0.2 (Table 3). This should be considered when using reference region quantification methods in the future study of SC PET imaging.

Although the results presented in this study are somewhat encouraging, there is additional work that can be done to validate or improve our findings. For example, ex vivo experiments may be completed to confirm SV2A density throughout the cervical, thoracic or full SC. Our group has completed preliminary Western blotting experiments to investigate the concentration of SV2A in the cSC compared to SV2A in gray matter regions of one 13 y.o. male rhesus monkey. The resulting SC/brain SV2A ratio, equivalent to a *V*_S_ ratio (assuming no difference in *K*_d_), was about 0.057. Given that SV2A density may be up to fivefold lower in the GM of the SC compared to that of the cortex, and that only 25% of the SC is comprised of GM, this ratio strongly agrees with an estimated relative SV2A concentration in the SC of 0.2(0.25) = 0.05. Using the *V*_S_ estimates from our brain imaging data reported in Tables 1 and 2, we report a SC/brain *V*_S_ ratio of approximately 0.056. Further studies are needed to explore if, and to what extent, SV2A changes throughout the length of the spinal cord.

SV2A image quality in the SC may be enhanced by improving PET scanner resolution. Given the dimensions of the SC, a PET system with ~ 3 mm resolution or better is crucial for successful imaging of the SC. SV2A PET radiotracers labeled with fluorine-18, such as [^18^F]SynVesT-1 [31], will result in higher quality PET images due to lower statistical noise. Furthermore, new radiotracers with less metabolism, or radiotracers with affinity greater than that of [^11^C]UCB-J(*K*_d_ <  ~ 3 nM) may also improve the quantification of SV2A PET in the spinal cord. As described above, the *B*_max_ in the full SC is expected to be ~ 20-fold lower than the cerebral cortex. To get a more useful measure of *BP*_ND_ (i.e., ~ 5 times greater than the *BP*_ND_ estimates of ~ 0.45 in the current study), the *K*_d_ of an SV2A radioligand should be at least 5 times lower than that of [^11^C]UCB-J (assuming no change in nondisplaceable uptake). Furthermore, additional radioligands that target other proteins or physiological functions of interest aside from SV2A, such as the serotonin transporter with [^11^C]AFM [32] or the 18 kDa translocator protein (TSPO) with [^11^C]PK11195 [33] or [^18^F]GE-180 [34], may be worthwhile in characterizing spinal cord pathophysiology.

## Conclusions

The current study suggests that PET imaging with [^11^C]UCB-J is a feasible way to measure synaptic density by SV2A in the human spinal cord. Results from simulation and human studies showed a consistent, albeit low, SV2A binding potential in the cord. Although future work is needed to validate specific binding and distribution of SV2A in the full SC, the results presented here show that imaging SV2A in the SC is possible. If validated, this imaging technique can be used to noninvasively evaluate the synaptic integrity of the SC in clinical populations including spinal cord injury, or spinal cord diseases like ALS, and may also be helpful in monitoring treatment progression.

## Supplementary Information

**Additional file 1**. Region of interest definition on HRRT PET Images - Top row: Sagittal view of early (0-10 mins) summed baseline (left) and blocking (right) [11C]UCB-J PET uptake images scaled to brain uptake (A) and scaled to spinal cord uptake (B). Bottom row: Cervical spinal cord ROI (cylinder of 3 voxel radius along 15 axial slices) is shown in green overlaid on the early PET image.

**Additional file 2**. Automated region of interest definition on mCI PET Images - (A) A single slice of a cropped anatomical CT image. (B) Binarized cropped CT image including voxels including vertebrae. (C) Closed, binarized cropped CT image including vertebrae and spinal cavity. (D) Difference image between (C) and (D) including spinal cavity. The Center of Mass in x- and y- directions was calculated on each axial slice of this image, and COMx and COMy were fit with a cubic spline. (E) A single slice of a continuous, full SC ROI mask.

## Data Availability

Code, data and material used and/or analyzed in this study can be made available by the corresponding author with reasonable request.

## Abbreviations

ALS: Amyotrophic lateral sclerosis
BBB: Blood brain barrier
*BP*_ND_: Binding potential in relation to the nondisplaceable compartment
BSCB: Blood spinal cord barrier
cSC: Cervical spinal cord
CT: Computed tomography
DVR: Distribution volume ratio
FOV: Field of view
GM: Gray matter
HRRT: High-resolution research tomograph
PET: Positron emission tomography
PVE: Partial volume effect
SC: Spinal cord
SCI: Spinal cord injury
SRTM2: Simplified reference tissue model 2
SV2A: Synaptic vesicle glycoprotein 2A
TAC: Time activity curve
tSC: Thoracic spinal cord
*V*_ND_: Nondisplaceable volume of distribution
*V*_S_: Specific volume of distribution; *V*_T_: Total volume of distribution
WM: White matter
