# Supplementary material for: Feasibility of imaging synaptic density in the human spinal cord using [11C]UCB-J PET
Source: EJNMMI Phys. 2022 May 3;9:32. doi: 10.1186/s40658-022-00464-0 (PMC9065222; doi:10.1186/s40658-022-00464-0)

Article Title: Feasibility of imaging synaptic density in the human spinal cord using [^11^C]UCB-J PET

Journal Name: European Journal of Nuclear Medicine and Molecular Imaging - Physics

Author Names: Samantha Rossano, Takuya Toyonaga, Jason Bini, Nabeel Nabulsi, Jim Ropchan, Zhengxin Cai, Yiyun Huang, Richard E. Carson

Affiliation and E-mail of Corresponding Author: Department of Radiology and Biomedical Imaging, Yale PET Center, Yale School of Medicine, New Haven, CT and Department of Biomedical Engineering, Yale University, New Haven, CT; [samantha.rossano@yale.edu](mailto:samantha.rossano@yale.edu)

**Additional File 1. Region of Interest Definition on HRRT PET Images –**

Top row: Sagittal view of early (0-10 mins) summed baseline (left) and blocking (right) [^11^C]UCB-J PET uptake images scaled to brain uptake (A) and scaled to spinal cord uptake (B). Bottom row: Cervical spinal cord ROI (cylinder of 3 voxel radius along 15 axial slices) is shown in green overlaid on the early PET image.


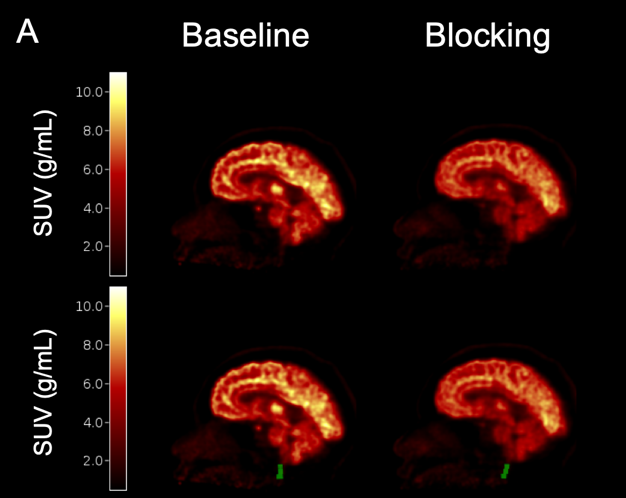

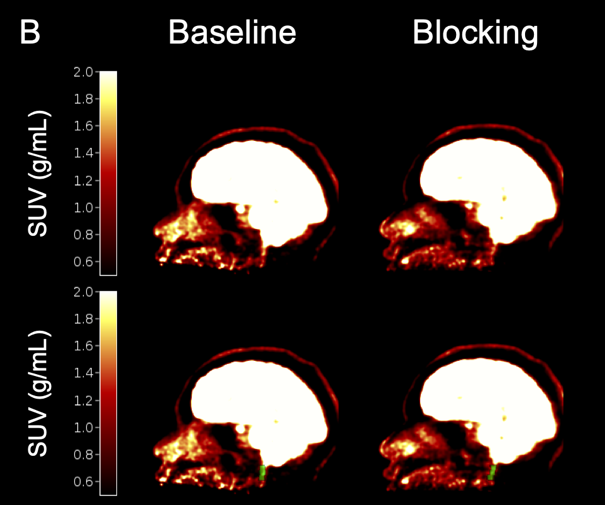

Supplement: Supplementary file 1 — Additional file 1. Region of interest definition on HRRT PET Images - Top row: Sagittal view of early (0-10 mins) summed baseline (left) and blocking (right) [11C]UCB-J PET uptake images scaled to brain uptake (A) and scaled to spinal cord uptake (B). Bottom row: Cervical spinal cord ROI (cylinder of 3 voxel radius along 15 axial slices) is shown in green overlaid on the early PET image. [file 40658_2022_464_MOESM1_ESM.docx]
