# Supplementary material for: Feasibility of imaging synaptic density in the human spinal cord using [11C]UCB-J PET
Source: EJNMMI Phys. 2022 May 3;9:32. doi: 10.1186/s40658-022-00464-0 (PMC9065222; doi:10.1186/s40658-022-00464-0)

Article Title: Feasibility of imaging synaptic density in the human spinal cord using [^11^C]UCB-J PET

Journal Name: European Journal of Nuclear Medicine and Molecular Imaging - Physics

Author Names: Samantha Rossano, Takuya Toyonaga, Jason Bini, Nabeel Nabulsi, Jim Ropchan, Zhengxin Cai, Yiyun Huang, Richard E. Carson

Affiliation and E-mail of Corresponding Author: Department of Radiology and Biomedical Imaging, Yale PET Center, Yale School of Medicine, New Haven, CT and Department of Biomedical Engineering, Yale University, New Haven, CT; [samantha.rossano@yale.edu](mailto:samantha.rossano@yale.edu)

**Online Resource 2. Automated Region of Interest Definition on mCT PET Images –**

(A) A single slice of a cropped anatomical CT image. (B) Binarized cropped CT image including voxels including vertebrae. (C) Closed, binarized cropped CT image including vertebrae and spinal cavity. (D) Difference image between (C) and (D) including spinal cavity. The Center of Mass in x- and y- directions was calculated on each axial slice of this image, and COMx and COMy were fit with a cubic spline. (E) A single slice of a continuous, full SC ROI mask.


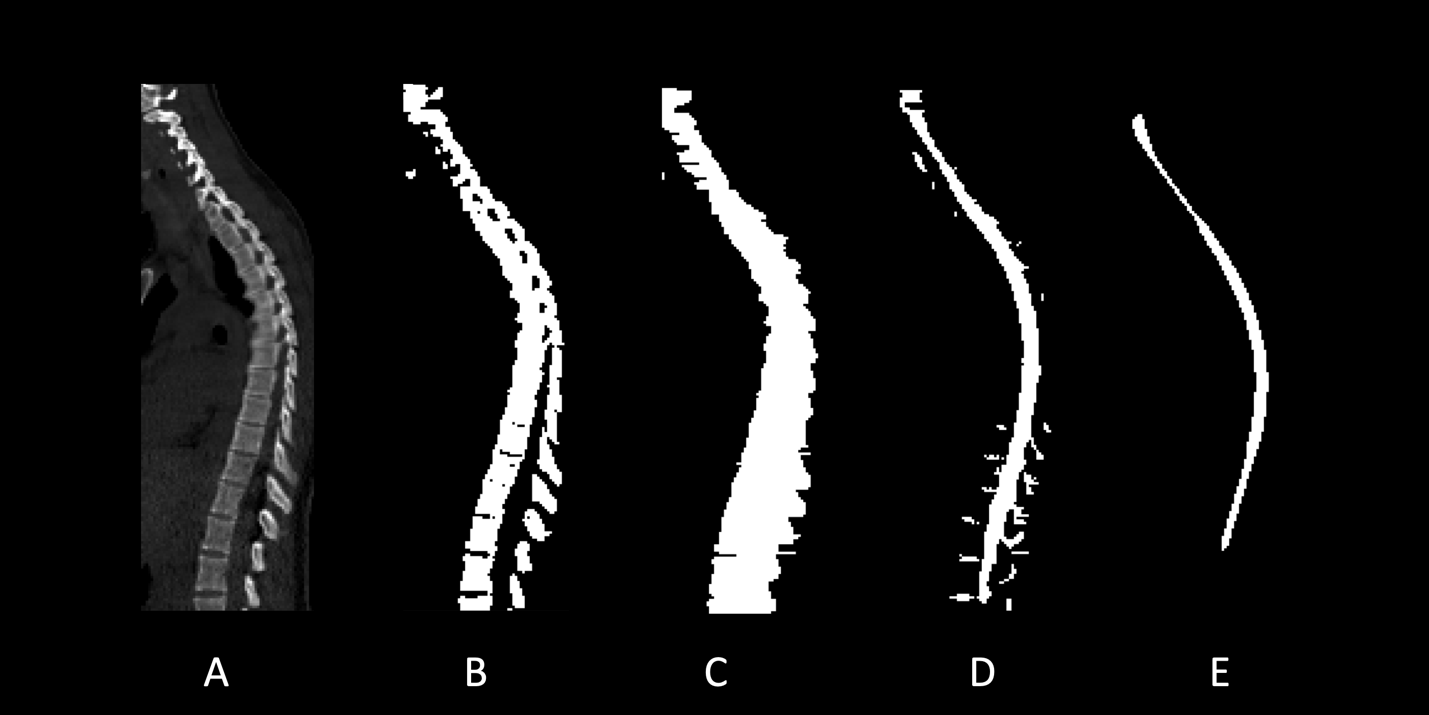

Supplement: Supplementary file 2 — Additional file 2. Automated region of interest definition on mCI PET Images - (A) A single slice of a cropped anatomical CT image. (B) Binarized cropped CT image including voxels including vertebrae. (C) Closed, binarized cropped CT image including vertebrae and spinal cavity. (D) Difference image between (C) and (D) including spinal cavity. The Center of Mass in x- and y- directions was calculated on each axial slice of this image, and COMx and COMy were fit with a cubic spline. (E) A single slice of a continuous, full SC ROI mask. [file 40658_2022_464_MOESM2_ESM.docx]
